# The Adsorption Behaviors of CO and H_2_ to FeO onto CaO Surfaces: A Density Functional Theory Study

**DOI:** 10.3390/molecules28165971

**Published:** 2023-08-09

**Authors:** Ziming Wang, Yaqiang Li, Yaping Dou, Kejiang Li, Wanhai Yu, Pengcheng Sheng

**Affiliations:** 1 Department of Automotive Engineering, Hebei Vocational University of Technology and Engineering, Xingtai 054000, China; 2 Hebei Special Vehicle Modification Technology Innovation Center, Xingtai 054000, China; 3 School of Metallurgical and Ecological Engineering, University of Science and Technology Beijing, Beijing 100083, China

**Keywords:** density functional theory, adsorption, H_2_ and CO, FeO, CaO surface

## Abstract

The adsorption behaviors of CO and H_2_ to FeO onto CaO surfaces have been studied using the density functional theory (DFT) to determine the reactions of FeO by CO and H_2_. The adsorption mechanisms of FeO clusters on the CaO(100) and CaO(110) surfaces were calculated first. The structure of the Ca(110) surface renders it highly chemically reactive compared with the Ca(100) surface because of low coordination. After gas adsorption, CO bonds to the O atom of FeO, forming CO_2_ compounds in both configurations through the C atom. H_2_ favors the O atom of FeO, forming H_2_O compounds and breaking the Fe-O bond. Comparing the adsorption behavior of two reducing gases to FeO on the Ca surface, the reaction of the CO molecule being adsorbed to generate CO_2_ compounds is exothermic. The reaction of H_2_ molecule adsorption to generate H_2_O compounds is endothermic. This property is essential for the inertial-collision stage of the reduction. However, the dissociation of the CO_2_ compound from the reaction interface will overcome a high energy barrier and slow down the reduction. The H_2_O compound dissociates from the surface more easily, which can accelerate the reduction.

## 1. Introduction

A large amount of carbon dioxide is emitted by the carbon reduction of iron ore in the ironmaking process. The steel industry faces enormous environmental pressures. The dominant blast furnace ironmaking process in the steel industry relies on carbon-based fuels for energy and reducing agents, resulting in large amounts of CO_2_ emissions into the environment. A key area for scientific research is the replacement of carbon with hydrogen in green metallurgical technology. The hydrogen-enriched reduction process can use a high percentage of hydrogen to reduce iron ore to metallic iron. The product is H_2_O, which reduces the CO_2_ gas generated by carbon-based reduction [1,2,3,4,5,6]. The contradiction between the increased requirements for iron ore quality and the deterioration of iron ore quality in the hydrogen-enriched reduction process is becoming increasingly prominent. This is a common problem that the current ironmaking process must face. An enhanced understanding of iron oxides in the hydrogen-based reduction process is the basis for solving this problem.

There are a wealth of studies dedicated to developing an experimental understanding of the iron oxide reduction behaviors of CO and H_2_ [7,8]. Bahgat et al. [9] explored the reduction of Fe_3_O_4_ single crystals by H_2_ and measured an activation energy of 99 kJ mol^−1^. The reaction rate slowed down at 900 and 950 °C at 83% and 89% reduction, respectively, indicating rate limitation by solid-state diffusion. Chen [10] demonstrated the isotherm reduction of Fe_2_O_3_ with a fluidized bed and measured the activation energy required for the reduction to FeO and Fe at 83.6 and 80.4 kJ mol^−1^, respectively. Bondale [11] explored the isotherm reduction of Fe_2_O_3_ pellets with H_2_/H_2_–CO mixtures. It was found that the reduction with pure H_2_ was faster compared with CO and gas mixtures and both the restrictions arising from the decrease in CO content as well as those related to gas diffusivity were revealed. Kim et al. [12] investigated the kinetics of cylindrical compacts of magnetite (Fe_3_O_4_) with H_2_ and H_2_–H_2_O (g) mixtures. The initial reduction rate increases with H_2_ partial pressure. However, as the reduction proceeds, an iron layer is formed and the rate decreases significantly. The thermodynamic analysis of the reduction shows that hematite (Fe_2_O_3_) reduction by CO and H_2_ leads to microstructural evolution [7,9]. Prior reports have indicated three steps: Fe_2_O_3_→Fe_3_O_4_→FeO→Fe. The reduction process leads to the formation of several cracks and holes in the process of microstructural transformation [13,14,15]. In addition, the control step of the generation of iron whiskers is the reduction of FeO to Fe. Moreover, when comparing the reduction behavior of CO and H_2_ gases on iron oxide, the reduction rate of H_2_ is higher than that of CO. The smaller size of the hydrogen atom favors the diffusion of the reducing gas, leading to a kinetically favorable reduction [16]. The current understanding of hydrogen-based direct reduction is limited to the macroscopic description and rate analysis of the reduction process. The microscopic reaction mechanism and electronic structure properties of iron oxides still lack scientific knowledge and evaluation. The mechanism of the competitive reduction of iron oxide by CO and H_2_ is not clear. Previous studies have demonstrated that the addition of CaO can control the basicity of blast furnace slag [17]. Meanwhile, CaO covers the surface of iron ore powder to increase the separation effect [18] and avoid the sponge iron surface reduction generated by the iron whiskers and thus leads to the sticking problem in the fluidized bed reduction process [19,20]. However, the conversion of the CaO and Fe_2_O_3_/FeO phase to the CaO–FeO phase at their interface is confirmed using SEM/EDS measurements. FeO is sensitive to CO and H_2_; therefore, the CaO–FeO phase behavior and reduction is interesting. Zhao et al. [21] investigated the reduction properties of Fe_2_O_3_ with different CaO contents in a CO atmosphere using thermogravimetric analysis. This study suggested that the doped Ca catalyzes the reduction of Fe_2_O_3_ by destroying the crystal structure and facilitating the diffusion of O_2_, especially as the decomposition of the FeO phase into Fe occurs [22]. The protective effect of CaO is reduced.

The density functional theory (DFT) has been used to explore the reduction of iron oxides. Zhong et al. [16] investigated the adsorption behavior of CO and H_2_ on the surface of FeO using DFT calculations to explore the reduction mechanism. The CO molecule binds to the O atom of the FeO surface to generate a C–O bond to produce CO_2_. When H_2_ adsorbs to the surface of FeO(110), the H_2_ molecule dissociates, forming an O–H bond and an Fe–H bond. For the adsorption of H_2_ onto the FeO(111) surface, H_2_ adsorbs to the O top of the FeO(111) surface and H_2_O is generated. In addition, Zhong [23] used DFT to show that CaO and MgO surfaces promote the binding of FeO to CO. Dong et al. [24] used thermogravimetric analysis (TGA) experiments and DFT to investigate the reduction of iron oxide, and the results indicate that the reduction of Fe^2+^ to Fe is the control step. Sun et al. [25] studied the adsorption of H_2_ on the Fe_3_O_4_ surface and showed that H atoms are more likely adsorb to the O atom on the surface of Fe_3_O_4_ rather than to Fe atoms. Zhong used DFT theory to investigate the promotion of CaO and MgO surfaces due to their relatively larger binding energies for the binding of CO to FeO.

The experimental results were used to study the thermodynamics and kinetics involved in the reduction of iron oxide with CO and H_2_, explaining the limiting step in the reaction. However, in practice, the competitive reduction mechanism of iron oxide by CO and H_2_ is not clear. More than one single CaO surface can take part in the reduction of iron oxide processes. An atomistic insight into reducing agents’ absorption and the dissociation of product on the CaO surface is desirable. Here, theoretical work with the DFT was dedicated to study the reactions of FeO by CO and H_2_ on CaO surfaces on the atomic scale.

## 2. Results and Discussion

### 2.1. Adsorptions of FeO to CaO Surfaces

Recent studies [26,27] have shown that iron oxide can be represented by clusters of FeO, Fe_2_O_2_, Fe_3_O_4_, and Fe_2_O_3_ to study the mechanism of its reduction by CO. A single Fe–O bond is the basic structure of complex iron oxides. Therefore, we use the FeO cluster to illustrate the effect of CaO on the reduction of FeO by CO and H_2_.

**Figure 1:**
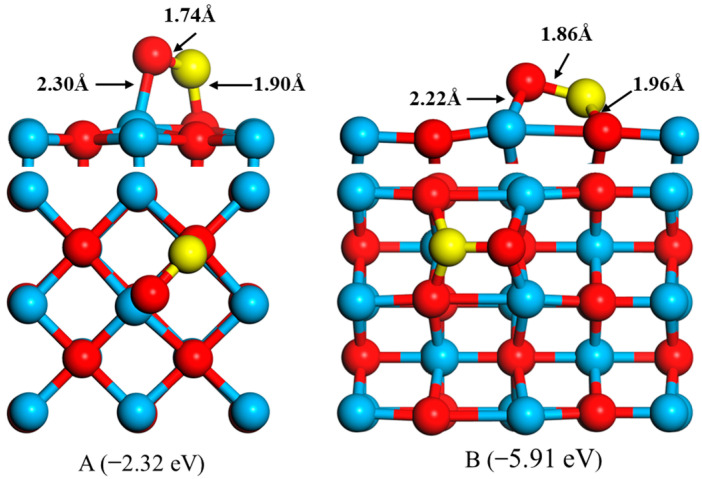
Top and side views of the optimized structure of FeO on the Ca(100) surface (**A**) and Ca(110) surface (**B**),where yellow, red, and blue balls represent the Fe, O, and Ca atoms, respectively.

The interaction of CO_2_ with the CaO(100) surface and the CaO(110) surface was calculated by placing the FeO cluster onto three adsorption sites: O top, Ca top, hollow, and bridge. The all-adsorption configurations were also tested. Considering the FeO clusters and the CaO surface structure, the Fe atom prefers to approach the O top site on the CaO surface, while the O atom of FeO is the most energetically favorable binding partner for the Ca atom. The optimized geometry of FeO adsorption onto the CaO surface is shown in Figure 1. At the interface, the bond distances of Fe–O and O–Ca are 2.30 Å and 1.90 Å, respectively. The binding energy of these configurations is −2.32 eV, respectively. Zhong showed that the bond lengths of the FeO clusters adsorbed on CaO(100) are 2.36 Å and 1.83 Å, with a binding energy of 2.58 eV. These values are comparable to those reported by Zhong [23]. The calculated Fe–O bond length on the CaO(100) and Ca(110) surfaces are 1.74 Å and 1.86 Å, respectively, which are longer than the Fe–O bond length (1.70 Å) in the free FeO cluster. The binding energy of B is −5.91 eV. Large negative binding energies were obtained for the Ca(110) surface indicating more stable complexes because of the low coordination of the top adsorption site.

### 2.2. Adsorptions of CO and H_2_ to FeO onto the CaO Surfaces

**Figure 2:**
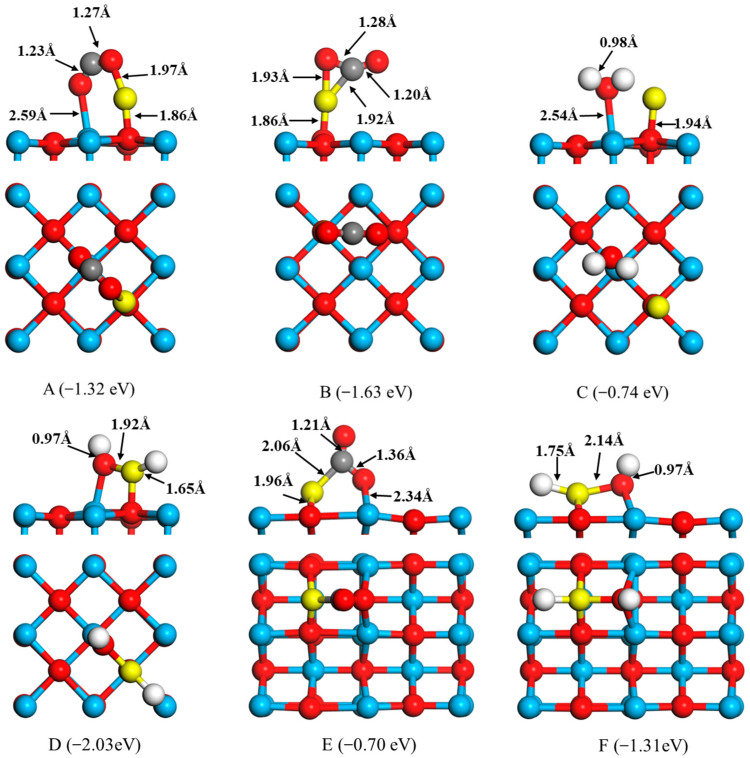
Optimized structures of CO and H_2_ interaction with FeO the surfaces of CaO(100) (**A**–**D**) and CaO(110) (**E**,**F**), where yellow, red, white, and blue balls represent the Fe, O, H, and Ca atoms, respectively.

**Figure 3:**
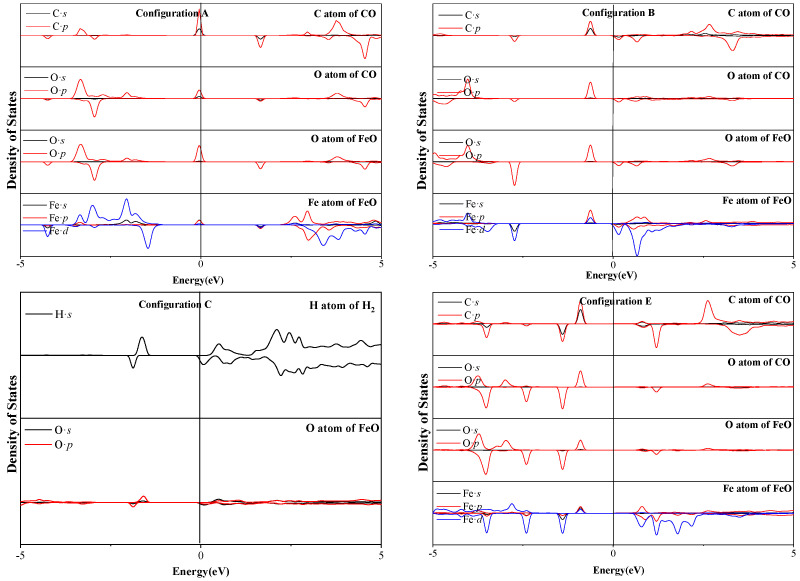
PDOS of adsorption configurations A, B, C, and E in Figure 2. The Fermi level is located at 0 eV.

Stable adsorption configurations and the corresponding adsorption energies of CO and H_2_ to FeO onto the CaO surfaces are shown in Figure 2. In configuration A in Figure 3, the CO is captured by the FeO cluster, breaking the Fe–O bond and forming a new C–O bond (1.23 Å). The O of the CO adsorbs to the Fe atom (1.97 Å). For structure B, the C atom can move closer to the O and Fe atoms, forming new C–O and C–Fe bonds with bond lengths of 1.91 Å and 1.20 Å, respectively. At the same time, the O–Fe bond is broken and the C−O bond length of the CO is stretched to 1.28 Å. Our calculations predict that configurations A and B of the binding energies are −1.32 eV and −1.63 eV, respectively, which indicates considerable interaction between CO and FeO. The bonding of H_2_ to FeO on the surface of CaO(100) is illustrated in Figure 2C,D. In configuration A, adsorption of the H_2_ molecule leads to a non-negligible change in the FeO geometry, that is, the O–Fe bond of the FeO molecule is broken and the O atom is bound to two H atoms to form H_2_O compounds. The bond length between H and O, 0.98 Å, is similar to the distance between two atoms in a free water molecule (0.96 Å). For structure D, the H_2_ is dissociated and adsorbed to form a new H–O bond and H–Fe bond with bond lengths of 0.97 Å and 1.65 Å, respectively. The O–Fe bond is extended to 1.92 Å. The binding energies of configuration C and D are −0.74 eV and −2.03 eV. Figure 2E,F shows the binding of CO and H_2_ with FeO on the CaO(110) surface. In configuration E, CO molecules are adsorbed to form a C–O bond (1.36 Å) and C–Fe bond (2.06 Å), in which the adsorption energy is −0.70 eV. The distance between the O and Fe atoms extended to 2.78 Å and the O–Fe bond is broken. The adsorption of H_2_ to FeO on CaO(110) surface is illustrated in Figure 2F. H_2_ adsorbs to FeO, breaking the H–H bond and forming a new H–O bond (0.97 Å) and a new H–Fe bond (1.75 Å). The binding energy is −1.31 eV.

### 2.3. Partial Density of States

The adsorption of CO/H_2_ onto FeO to generate surface active compounds is the crucial step for reduction. Considering the structures of adsorbate and adsorbent, three CO–FeO configurations (C bond to the O atom of FeO forming CO_2_ compounds) and one H_2_–FeO configuration (H bond to the O atom of FeO forming H_2_O compounds) were explored to investigate the interaction mechanism. The partial state density (PDOS) of configurations A, B, C, and E were calculated, as shown in Figure 3.

In configurations A, B, and D of Figure 3, after the adsorption of CO on the FeO configuration, C 2s, C2p, O 2s, and O 2p of FeO shift to lower energies below the Fermi energy level and overlap in the range of −5 eV to −0.04 eV, indicating stronger interactions between them. The appearance of these overlapping peaks is related to the formation of the CO_2_ compound. In addition, it is clear that a strong resonance between the Fe 3d orbital peak and the C 2s and 2p orbital peaks is related to the formation of the C–Fe bond in configuration B and E. In configuration C, H 1s and O 2p overlap at energies near −1.9 and −1.6 eV, suggesting that s–p hybridization forms covalent bonds. In addition, there are some resonance peaks, which occur at energies above the Fermi level, between the C 2p and the O 2p for all these configurations, indicating the antibonding interactions between the C atoms and the O atoms. Combining structural analysis and electronic properties, CO and H_2_ is chemisorbed on O of FeO.

### 2.4. Charge Transfer

**Figure 4:**
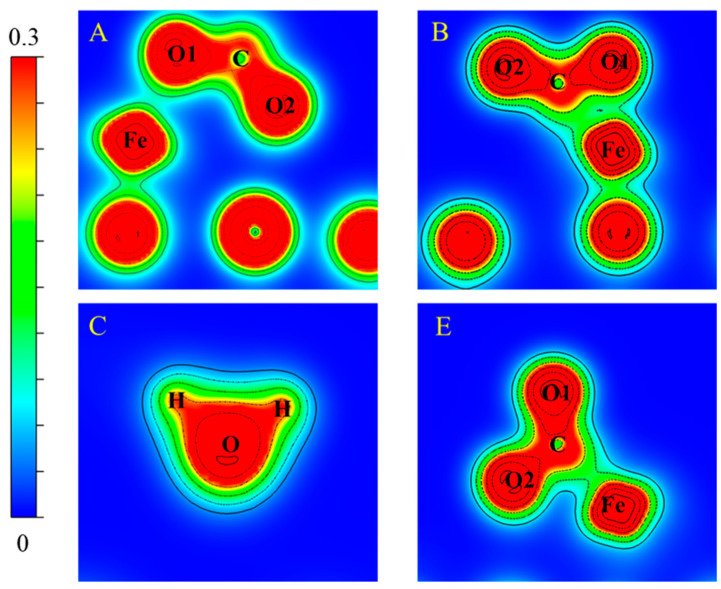
Bonding charge density for adsorption configurations A, B, C, and E in Figure 2.

Figure 4 shows that the Löwdin charge analysis of CO molecules or H_2_ molecules bond to FeO on the Ca(100) surface. For configuration A, the Löwdin populations for O(1), C, Fe, and O(2) are 2s^1.62^2p^4.72^, 2s^0.99^2p^2.50^, 4p^0.51^3d^6.58^4s^0.67^, and 2s^1.64^ 2p^4.70^, respectively, while the atomic population distribution before CO adsorption are 2s^1.64^2p^4.45^, 2s^1.64^2p^2.10^, 4p^0.66^3d^6.38^4s^0.51^, and 2s^1.74^2p^4.91^, respectively. Changes in population for the 3d orbital and 4s orbital of Fe enable the acceptance of electrons after adsorption, which shows that there are interactions between CO and Fe atoms. For configuration A and similarly for configuration B, the Löwdin population for Fe is 4p^0.79^3d^6.46^4s^0.59^, which shows that Fe accepts electrons after adsorption. In configuration D in Figure 4**,** the Löwdin populations for O(1), C, Fe, and O(2) are 2s^1.67^2p^4.68^, 2s^0.99^2p^2.64^, 4p^0.86^3d^6.33^4s^0.54^, and 2s^1.67^2p^4.68^, respectively, while the atomic population distributions before CO adsorption are 2s^1.64^2p^4.45^, 2s^1.64^2p^2.10^, 4p^0.83^3d^6.30^4s^0.46^, and 2s^1.73^2p^5.12^, respectively. The 4p orbital, 3d orbital, and 4s orbital of Fe accept electrons after adsorption, while the populations of the C 2s orbital and the 2p orbital are decreased. Therefore, FeO and CO act as electron acceptors and electron donors in the FeO-CO bond system, respectively. The rearrangement of electrons after adsorption indicates that a covalent bond is formed between CO and FeO.

For the H_2_–FeO system (configuration C), the population of H and O is 1s^0.62^. The rearrangement of electrons is due to the transfer of electrons from the H atom to the O atom, forming two H–O bonds. This chemical adsorption of the O atom by H_2_ produces compounds of H_2_O.

### 2.5. Reaction Path of FeO by CO and H_2_ Molecule

**Figure 5:**
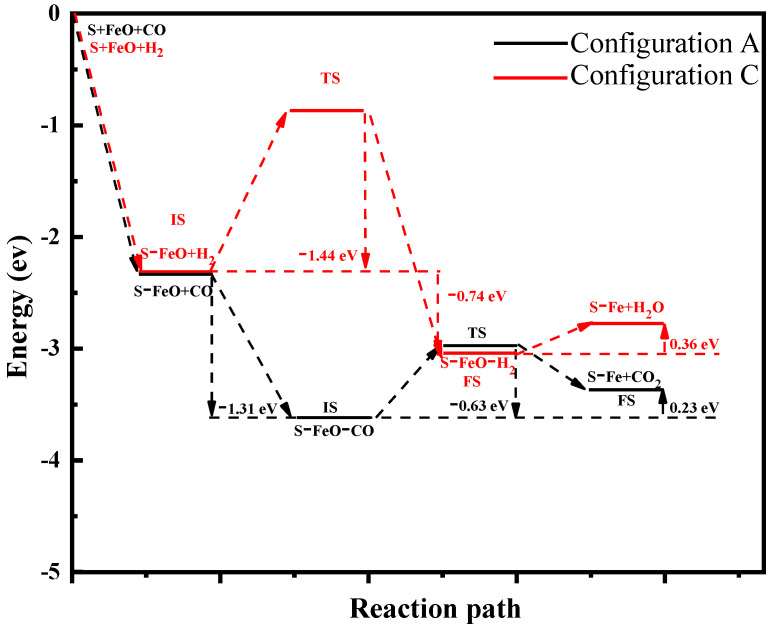
Calculated energy profiles for the reduction path of FeO by CO and H_2_ on CaO(100) surface. S-FeO + CO/H_2_: state of FeO cluster is adsorbed on the Ca(100) surface and free CO/H_2_ molecule; S-FeO-CO/H_2_: state of CO/H_2_ is adsorbed by the FeO; S-Fe-CO_2_/H_2_O: state of CO_2_/H_2_O compounds generated; S-Fe + CO_2_/H_2_O: state of dissociation of CO_2_/H_2_O gas.

**Figure 6:**
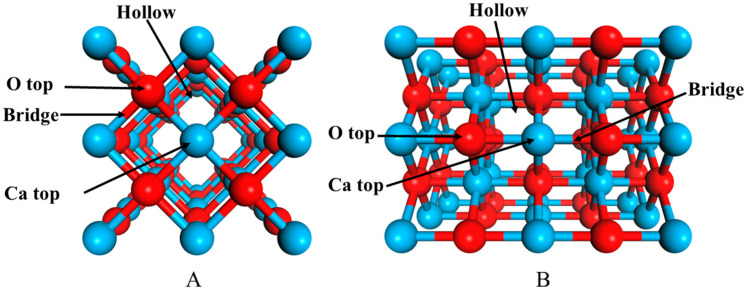
Top views of the optimized CaO surfaces: (**A**) CaO(100) surface; (**B**) CaO(110) surface, where the blue ball represents the Ca atom and the red ball represents the O atom.

The reaction path of FeO by CO and H_2_ molecule on the CaO(100) surface can be described as the following process, involving sequential steps: firstly, FeO clusters are adsorbed on the Ca(100) surface to form new Fe–O bonds and O–Ca bonds (S–FeO+ CO/H_2_ state) with a binding energy of 2.32 eV (Figure 5). In the process of the reduction of FeO by CO, the CO molecule is adsorbed to generate CO_2_ compounds (S–FeO–CO state). The reduction process of FeO is similar to the ones reported for the FeO cluster on the CaO(100). Subsequently, The S–FeO–CO (IS) needs to cross through the 0.63 eV barrier to S–FeO–CO_2_(TS), and CO_2_ gas is then dissociated in an exothermic process (0.40 eV), as shown in Figure 6. In the reduction of FeO by H_2_ (Figure 5), the O–Fe bond of the FeO cluster is broken and the O atom is bound to two H atoms, crossing the energy barrier of 1.63 eV to experience an endothermic process to form the H_2_O compounds (S–Fe–H_2_O state). Finally, the H_2_O compound experiences an endothermic process (0.36 eV) to form the H_2_O gas (S–Fe+ H_2_O state).

Comparing the adsorption behavior of two reducing gases with FeO on the CaO(100) surface, the binding energy of the CO molecule is more negative and H_2_ needs to cross through a barrier to absorb FeO. The CO molecule is adsorbed to generate CO_2_ compounds in an exothermic reaction. The H_2_ molecule is adsorbed to generate H_2_O compounds in an endothermic reaction, indicating that the CO molecule is more easily captured than the H_2_ molecule. This property is essential for the inertial–collision stage of the reduction. Secondly, the energy barrier for the CO_2_ compound’s dissociation from the substrate indicates the greater difficult posed by dissociation. In the later stage of the reduction, the reaction is limited by both interfacial chemical reactions and gas diffusion. H_2_ has an advantage at this stage because the product of H_2_ dissociates from the FeO more easily than that of CO. In addition, the reduction of iron oxide by CO and H_2_ is the gas–solid heterogeneous reaction. Initially, the reactant gas reacts with the external surface and moves progressively inside incrementally. Compared with CO_2_ molecules, the smaller diameter of H_2_O molecules enhances diffusion and separation towards the interior of iron oxide, thereby accelerating the reaction. Our results reveal that the reduction of FeO by H_2_ is controlled by adsorption steps. High temperature has a positive effect on the adsorption of H_2_. In addition, the separation of CO_2_ and H_2_O from the reaction interface leads to the conversion from FeO to metallic iron, which is the final step of the iron oxide reduction reaction. CaO promotes the reduction of FeO into Fe by H_2_ at high temperatures, and then metallic iron accumulates to form iron whiskers.

## 3. Materials and Methods

Our first principles calculations were performed using the Quantum ESPRESSO package, adopting the spin–polarized plane-wave density functional theory [28]. The electronic structure was modeled by Generalized Gradient Approximation (GGA) with the Perdew–Burke–Ernzerhof (PBE) functional. Projector Augmented Wave (PAW) was employed to represent the interactions between the electrons and the ions. The cutoff energy was 50 Ry for the wavefunction and 500 Ry for the charge density [29]. The 4 × 4 × 1 Monkhorst–Pack grid was used for sampling the Brillouin zone. In order to realize the effect of the 3d state of Fe [30], the GGA + U method with U = 4.2 eV was used in all adsorption configurations [31,32]. For the FeO cluster, CO, and H_2_ optimization, the periodic slabs were decoupled using a vacuum layer (15 Å) to relax_._ The Ca(100) surface and the Ca(110) surface were modeled using periodic slabs of about five atomic layers with a 2 × 2 supercell, as shown in Figure 6. During optimization, the atoms in the bottom three layers of the CaO(100) surface and the CaO(110) surface were fixed, whereas the other layers and adsorbates were allowed to relax. The periodic slabs were decoupled using a vacuum layer (15 Å) to eliminate interaction between two adjacent slabs. The three bottom CaO(100) layers were fixed during relaxation. The optimized Ca–O bond length between the top two layers was 2.33 Å, which is approximately smaller than the experimental value of about 0.01%. Both configurations resulted in negligible changes in the geometry of the CaO(100) surface. The configuration of the fixed bottom three layers was reliable.

The following formula was used to calculate the adsorption energy of all possible configurations:

*E_ad_* = *E_sys_* − *E_surf_* − *E_mol_* (1)

where *E_sys_*, *E_mol_*, and *E_surf_* are the energy of the system, the isolated molecule, and the clean surface, respectively. All the computations were performed using the GPC supercomputer at the SciNet HPC Consortium on the Compute/Calcul Canada national computing platform.

## 4. Conclusions

The adsorption behaviors of CO and H_2_ to FeO onto the CaO surfaces have been determined using density functional theory to study the reactions of FeO with CO and H_2_. For the adsorption of FeO onto CaO surfaces, the energy of the adsorption of FeO onto the CaO(110) surface is higher than that onto the CaO(100) surface. After CO adsorption, CO bonds to the O atom of FeO, forming CO_2_ compounds in both configurations through the C atom. In the adsorption of H_2_, the H_2_ adsorbs to FeO, breaking the H–H bond and forming a new H–O bond and a new H–Fe bond. In addition, H_2_ favors the O atom of FeO, forming an H_2_O compound on CaO surfaces and the Fe–O bond is broken.

Our results reveal the competitive adsorption behavior of FeO by CO and H_2_ on the CaO surface. The CO molecule is adsorbed to generate CO_2_ compounds in an exothermic reaction. The H_2_ molecule is adsorbed to generate H_2_O compounds in an endothermic reaction. The results indicate that CO is more easily and stably adsorbed on the FeO than H_2_, which is essential for the inertial-collision stage of the reduction. However, the dissociation energy of the CO_2_ compound from the reaction interface will overcome a higher energy barrier than that of H_2_O. In the later stage of the reduction, the reaction is limited by both interfacial chemical reactions and gas diffusion. H_2_ has an advantage at this stage. Therefore, the reduction of FeO by H_2_ is controlled by adsorption steps. High temperature has a positive effect on the adsorption of H_2_. In addition, the separation of CO_2_ and H_2_O from the reaction interface leads to the conversion from FeO to metallic iron, which is the final step of the iron oxide reduction reaction. CaO promotes the reduction of FeO into Fe by H_2_ at high temperatures, and then metallic iron accumulates to form iron whiskers. Sticking can easily occur in the high temperature ironmaking reduction of fine iron ore, thereby decreasing the reduction efficiency.

## Data Availability

The data that support the findings of this study are available from the corresponding author upon reasonable request.
